# Identifying risk factors for sarcopenia using machine learning: insights from multimodal data

**DOI:** 10.1007/s41999-025-01245-5

**Published:** 2025-06-05

**Authors:** Felicita Urzi, Domen Šoberl, Ornella Caputo, Marco Narici

**Affiliations:** 1 Faculty of Mathematics, Natural Sciences and Information Technologies, University of Primorska, Koper, Slovenia; 2 Department of Biomedical Sciences, University of Padova, Padua, Italy

**Keywords:** Sarcopenia, Risk factors, Nutrition, Genetics

## Abstract

**Purpose:**

This study aims to identify key risk factors for sarcopenia using machine learning models, leveraging anthropometric, biochemical, functional, nutritional, and genetic data. By developing predictive models, the research seeks to improve early detection, enhance diagnostic accuracy, and facilitate personalized interventions for individuals at risk of sarcopenia.

**Methods:**

We analysed multimodal data from 484 older adults. Two scenarios: Set-a (including SARC–CalF, excluding SARC-F) and Set-b (including SARC-F, excluding SARC–CalF) were applied in a three-stage modeling process with progressively reduced features and optimized predictive performance using machine learning models. Key predictors were ranked using SHAP values, and model performance was evaluated using AUC, accuracy, sensitivity, and specificity. Internal validation and DeLong’s test were applied to assess robustness and statistical differences.

**Results:**

The most predictive risk factors included functional measures (chair stand, gait speed), nutritional indicators (protein, folate, copper, vitamin B7), clinical conditions (diabetes, comorbidities, low-density lipoprotein (LDL)), and anthropometric markers (body mass index (BMI), calf circumference). Genetic features also contributed to risk stratification. The best-performing model Set-b (with screening test SARC-F) achieved an AUC of 0.951 and an accuracy of 93.62%. While SARC–CalF showed higher individual feature importance, the model achieved an AUC of 0.945 and accuracy of 92.2%.

**Conclusions:**

This study highlights that traditional sarcopenia screening can be enhanced by capturing complex interplay of functional, nutritional, clinical, and genetic factors, offering clinicians a more accurate and tailored tool for early detection and risk stratification. Future research should focus on validating these models in larger, independent, and longitudinal cohorts to assess their predictive utility across diverse populations and over time.

**Supplementary Information:**

The online version contains supplementary material available at 10.1007/s41999-025-01245-5.

## Key summary points

**Aim:**

To identify the most relevant multimodal risk factors for sarcopenia and evaluate how SARC-F and SARC–CalF influence machine learning model performance and feature prioritization.

**Findings:**

Functional measures (chair stand, gait speed), nutritional indicators (protein, folate, copper, vitamin B7), clinical conditions (diabetes, comorbidities, low-density lipoprotein), anthropometric markers (body mass index, calf circumference), and genetic markers (MTHFR polymorphism, Sarcopenia Risk Genotype (SRG)) were among the strongest predictors of sarcopenia across models.

**Message:**

Machine learning approaches can uncover complex risk factor interactions and enhance sarcopenia screening by integrating functional, nutritional, clinical, and genetic data beyond traditional tools alone.

**Supplementary Information:**

The online version contains supplementary material available at 10.1007/s41999-025-01245-5.

## Introduction

Sarcopenia is a syndrome associated with progressive loss of muscle mass, strength and function, with negative consequences for physical performance, including falls, reduced mobility, frailty, and loss of independence [1]. The presence of sarcopenia also affects the course of many chronic diseases, which are among the main causes of death and disability [2]. The acquired knowledge about the causes and consequences of sarcopenia led to the official recognition of the condition as a muscle disease with an International Classification of Diseases (ICD-10CM) (M62.84) code [3]. Today, the most widely accepted definition and diagnostic criteria for sarcopenia are that proposed by the European Working Group on Sarcopenia in Older People (EWGSOP2) [1]. In the consensus paper EWGSOP2 recommends a case-finding (use of the SARC-F questionnaire) approach when a patient reports relevant symptoms of sarcopenia. These symptoms include falling, weakness, slow walking speed, difficulty rising from a chair, self-reported muscle wasting, and impaired activities of daily living.

**Fig. 1:**
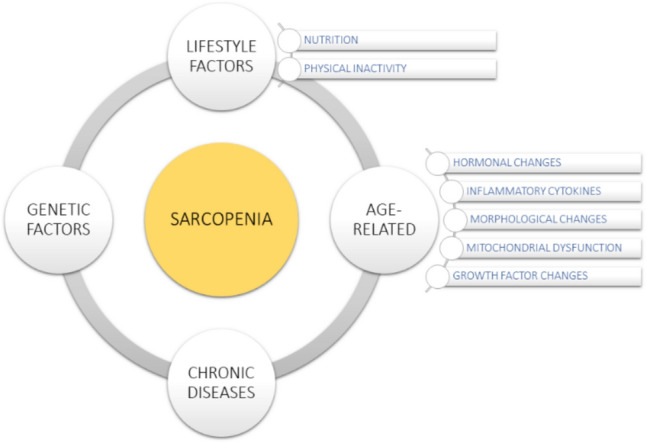
Factors contributing to sarcopenia

Sarcopenia is considered a geriatric syndrome, and it has been recognised that several factors contribute to its onset or progression (Fig. 1). Changes in muscle morphology, neurodegenerative processes, anabolic and sex hormone production or sensitivity, protein balance, increased oxidative stress, inflammation [4] and genetic predisposition [5] are important risk factors. In addition to these endogenous factors, inadequate nutrition and a sedentary lifestyle contribute to the complex etiology of sarcopenia [6].

Although diagnostic methods and tools for the assessment of sarcopenia have been defined and widely accepted [7], research into the underlying risk factors remains limited due to the multifactorial nature of the condition. Some contributors, such as acute illness or inactivity, can trigger rapid muscle loss, while others accumulate gradually over time. In many cases, sarcopenia results from a complex interplay of genetic, nutritional, metabolic and biochemical factors, making it difficult to pinpoint a single cause. Traditional methods may not fully capture these multidimensional interactions, which can lead to underdiagnosis or misclassification. Machine learning (ML) models, by contrast, offer the ability to analyse high-dimensional, heterogeneous data sets, integrating multiple variables to identify complex patterns and interactions that may be overlooked by conventional methods.

Different studies have addressed the issue of sarcopenia based on various ML models, using diverse feature sets. For instance, some models have utilized clinical and biological features to predict sarcopenia, incorporating data, such as muscle mass, strength, and biomarkers [8–10]. Other models have focused on physical activity [11], providing insights into muscle function and activity patterns. In addition, obesity-related features [12] and computed tomography (CT) [13] scans have been employed to capture adipose tissue distribution and muscle quality. Furthermore, genetic studies have identified sarcopenia-associated genes, which are integrated into ML models to enhance predictive accuracy [14]. Even though gene-based models have achieved a high level of accuracy, their use has been constrained by the high costs of gene analysis and limited integration into routine clinical testing.

The cause of sarcopenia is not yet fully understood, but thanks to the use of artificial intelligence, such as ML models, the process of diagnosis, uncovering the biological mechanism, and the development of precision medicine, have become easier, faster, and more accurate [15].

The present study aimed to identify the most relevant multimodal risk factors for sarcopenia and to evaluate how the inclusion of SARC-F and SARC–CalF influences machine learning model performance and feature prioritization. We further investigated whether machine learning approaches can enhance traditional sarcopenia screening tools by capturing complex, multidimensional interactions across diverse data types, thereby improving early detection and enabling more targeted prevention and intervention strategies.

## Materials and methods

### Studies (participants)

Data were collected from 484 older adult participants (30% of men; ages ranged from 65 to 97 years; average age of 76 years), comprising 52% independent-living individuals and 48% residents of nursing homes. Approximately 18% of the participants were identified as sarcopenic. Participants were recruited using convenience sampling from consecutive cross-sectional studies on sarcopenia [16–19] and relevant sarcopenia-related parameters were subsequently extracted. The participants underwent assessments, including physical examination, nutritional evaluation, body composition analysis and laboratory testing. Sarcopenia diagnosis was performed following the European Working Group on Sarcopenia in Older People (EWGSOP2) guidelines [1], with detailed protocols provided in Supplementary Text SI1. In addition, sarcopenia was screened using the SARC-F and SARC–CalF questionnaires (Text SI2). Habitual dietary intake was assessed using a 3-day weighed dietary record, covering two weekdays and one weekend day (Text SI3). Genetic risk assessment involved analyzing single nucleotide polymorphisms in four candidate genes and calculating a Sarcopenia Risk Genotype (SRG) score (detailed in Text SI4).

We collected data on 68 features including sarcopenia questionnaires, functional parameters (grip strength, gait speed, chair stand test), anthropometric features (body mass index, fat mass, total body water, skeletal mass index, calf circumference, visceral fat, bone density), dietary features, health features (COPD, diabetes, hypertension, heart disease, and depression), genetic features, and biochemical features(S-glucose, S-CRP, S-cholesterol, S-triglycerides, S-HDL, and S-LDL). Detailed descriptions of the features are shown in Supplementary Table SI1.

### Machine learning models

To investigate potential risk factors for sarcopenia, we compared the performance of machine learning (ML) models under two scenarios (Fig. SI1): one that includes the SARC–CalF test excluding SARC-F, with calf circumference embedded in the score (Set-a) and one that includes the SARC-F test, excluding SARC–CalF with calf circumference as an independent variable (Set-b). Both sets retained the same pool of additional functional, nutritional, clinical, and genetic features. By analysing models that include either SARC-F or SARC–CalF (but not both simultaneously) we aimed to: avoid multicollinearity from overlapping features; assess how each tool influences the selection and weighting of other predictors (e.g., nutritional, genetic, or biochemical variables); determine whether one tool led to more robust or interpretable models in a multimodal data set context. This approach allowed us to explore how the initial clinical screening method interacts with complex data sets and contributes to risk stratification. For both conditions, a three-stage modeling pipeline was applied:

#### Stage 1: Full feature modeling

In the first stage, we trained models using the full set of available features (Supplementary Table SI1) to predict sarcopenia. Five ML algorithms were evaluated: decision trees (DT), random forest (RF), support vector machine (SVM), gradient boosting (GB), and neural networks (NN). All models were implemented using the scikit-learn Python library. For RF and GB, we set n_estimators = 100. For SVM, a linear kernel was used. The Neural Network model was configured as a Multilayer Perceptron (MLP) with two hidden layers containing 200 and 100 neurons, respectively. Hyperparameters were fine-tuned experimentally using tenfold cross-validation on the training set. To statistically compare model performances, one-way ANOVA was applied to the classification accuracy (CA validation) scores obtained from the cross-validation.

#### Stage 2: Feature selection with SHAP

To identify the most informative predictors, we applied a model-agnostic feature selection approach based on SHAP (SHapley Additive exPlanations) values. SHAP values were computed for each of the five ML models trained in Stage 1. Features with non-zero SHAP values were considered important. A feature was retained for further analysis if it appeared with non-zero SHAP values in at least three out of the five ML models. The resulting scores (ranging from 0 to 5) are presented in Supplementary Tables SI5 and SI6. These scores represent the number of models that identified each feature as important. Based on these results, a reduced subset of features was selected, and new models were trained using the RF algorithm, which had demonstrated the highest performance in Stage 1.

#### Stage 3: Elbow-based feature optimization

To determine the optimal number of features, we plotted a performance curve (elbow plot) showing classification accuracy as a function of the number of top-ranked features. The elbow point at which adding more features resulted in diminishing returns in accuracy was used to identify a stable feature count. The analysis revealed that the top 15 features captured the most predictive information. This reduced set was used for final model training with RF, striking a balance between predictive accuracy, model simplicity, and interpretability.

#### Statistical comparison of model performance

To statistically assess differences in model performance across stages, we employed two complementary evaluation methods. DeLong’s test was used to compare the area under the receiver operating characteristic curves (AUCs) between models at different stages within each feature set (Set-a and Set-b). This non-parametric test, implemented using a custom Python-based procedure, allows comparison of correlated ROC curves derived from the same test data set. It evaluates whether observed differences in AUCs are statistically significant. McNemar’s test was applied to assess whether the classification differences between models were statistically significant in terms of accuracy. This test, suitable for paired nominal data, was conducted on contingency tables of predictions from two models evaluated on the same test set. McNemar’s exact *p* values were computed using the stats-models Python package. All performance metrics, including AUC, accuracy, sensitivity, specificity, and F1 score, were calculated using the scikit-learn library. In addition, 95% confidence intervals for AUCs were estimated using a bootstrap method with 1,000 iterations to assess the stability and variability of model performance. A significance threshold of *p* < 0.05 was used for all statistical tests.

#### Handling the imperfect data set

Due to the limited size of our data set and the absence of publicly available external databases with matching variables and population characteristics, external validation could not be performed. Instead, we used an internal validation approach by splitting the data set into training (70%) and testing (30%) sets using stratified sampling to preserve class distribution. The data set exhibited significant class imbalance, with non-sarcopenic individuals representing 81.7% of the population. To address this, we applied the synthetic minority over-sampling technique (SMOTE) to artificially balance the classes within the training set. This helped enhance the model’s sensitivity to sarcopenia cases.

To handle missing values, we employed advanced model-based imputation techniques. Numerical features were imputed using a Random Forest Regressor, while categorical features were imputed using a Random Forest Classifier. These methods utilized feature relationships to provide more accurate estimates than simple mean or mode imputation. Furthermore, to enhance the robustness of our evaluation, we applied cross-validation on the training set. This approach ensured that performance metrics were not overly dependent on a specific data split and provided a more reliable estimate of model generalizability.

#### Outlier detection and normalization

To ensure comparability among features and mitigate the influence of scale, all numerical attributes were normalized prior to analysis. Following normalization, we applied the z-score method to identify potential outliers. An instance was considered an outlier if its absolute z-score exceeded 3 (i.e., ∣z∣ > 3). Based on this criterion, 14 instances were identified as outliers (which represents 2.89% of the total population) and subsequently removed from the data set to enhance data quality and reduce noise in the analysis.

## Results

The average age of participants in our sample was 76 years, with 30% being men. Based on the EWGSOP2 criteria, 18% of participants were classified as sarcopenic. When screening tests were applied, SARC–CalF identified 18% as sarcopenic, while SARC-F identified 25%. Baseline characteristics and proportions of participants by living environment are detailed in Supplementary Table SI2. Participants from nursing homes had a significantly higher average age compared to those independently living. The nursing home group also demonstrated a higher prevalence of sarcopenia, lower grip strength and gait speed, and higher body mass index (BMI) and total body water content (*p* < 0.01). In addition, nursing home residents had significantly higher rates of hypertension and mild cognitive impairment (*p* < 0.01), as illustrated in Supplementary Figs. SI2 and SI3.

**Fig. 2:**
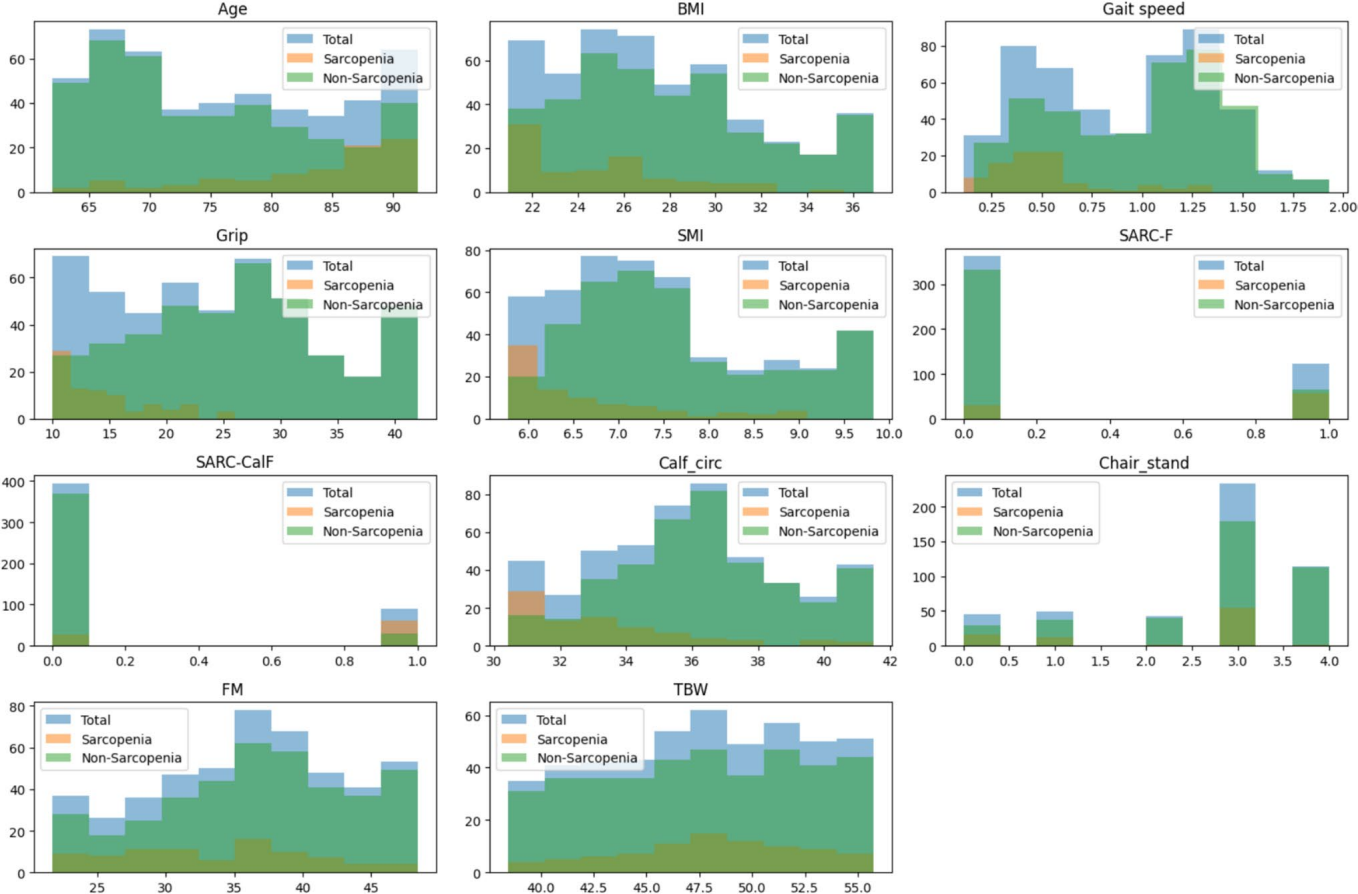
Distribution and comparison of baseline characteristics in the total sample, pooled group with sarcopenia, and non-sarcopenic group. *Note*: BMI, body mass index; SMI, skeletal muscle index; FM, fat mass; TBW, total body water

A comparison of baseline characteristics between participants with and without sarcopenia (classified according to EWGSOP2) is presented in Fig. 2 and Table SI3. Participants with sarcopenia were significantly older and had lower grip strength, skeletal muscle index (SMI), calf circumference, gait speed, BMI, and total body fat mass compared to those without sarcopenia (*p* < 0.01). Moreover, a higher proportion of sarcopenic participants were female. Sarcopenic individuals also exhibited higher proportions of multiple chronic diseases commonly associated with sarcopenia (Figs. SI4, and SI5).

### Performance of machine learning models under two scenarios (Set-a and Set-b)

Given these observed differences, we proceeded to investigate potential risk factors for sarcopenia by comparing the performance of machine learning models under two scenarios [Set-a (Excluding SARC-F) and Set-b (Excluding SARC–CalF)] using a structured, three-stage modeling pipeline.

#### Stage 1: Full feature modeling

Models trained on Set-a showed strong performance. Among these, the random forest (RF) algorithm performed best, achieving a test classification accuracy of 91.49%, with a sensitivity of 68.00%, specificity of 96.55%, F1 score of 0.739, and an area under the curve (AUC) of 0.92. The gradient boosting (GB) model also demonstrated strong predictive capability, with a test accuracy of 88.65%, sensitivity of 76.00%, specificity of 91.38%, F1 score of 0.704, and AUC of 0.91. Support Vector Machines (SVM) yielded comparable results, with a test accuracy of 87.94%, sensitivity of 72.00%, specificity of 91.38%, F1 score of 0.679, and AUC of 0.91. The decision tree (DT) algorithm showed moderate performance, achieving a test accuracy of 85.11%, sensitivity of 76.00%, specificity of 87.07%, F1 score of 0.644, and AUC of 0.82. Neural networks (NN) had the lowest performance, with a test accuracy of 78.72%, sensitivity of 64.00%, specificity of 81.90%, F1 score of 0.516, and AUC of 0.75 (Table SI4).

The models on Set-b showed similar performance trends. The RF model achieved a test classification accuracy of 89.36%, with a sensitivity of 64.00%, specificity of 94.83%, F1 score of 0.681, and AUC of 0.92. The GB model also reached an accuracy of 89.36%, with a sensitivity of 68.00%, specificity of 93.97%, F1 score of 0.694, and AUC of 0.93. SVM maintained strong performance with a test accuracy of 87.94%, sensitivity of 72.00%, specificity of 91.38%, F1 score of 0.679, and AUC of 0.91. The DT model again showed moderate performance, with a test accuracy of 85.11%, sensitivity of 68.00%, specificity of 88.79%, F1 score of 0.618, and AUC of 0.78. NN continued to perform poorly, achieving a test accuracy of 78.72%, sensitivity of 28.00%, specificity of 89.66%, F1 score of 0.318, and AUC of 0.75 (Table SI5).

To determine whether there were statistically significant differences in performance among the machine learning models, a one-way ANOVA was conducted using accuracy values from cross-validation. Post hoc comparisons using the Tukey HSD test revealed no statistically significant differences (*p* > 0.05) between the top-performing models. Consequently, the model with the highest observed classification accuracy from each machine learning method was selected for further evaluation. For both Set-a and Set-b, the Random Forest algorithm consistently achieved the highest accuracy and was, therefore, chosen as the representative model for Stages 1 and 2 of the modeling pipelines.

#### Stage 2: Feature selection with SHAP

In Stage 2, we refined the feature set by including only those features that were identified as important (i.e., non-zero SHAP values) in at least 3 out of 5 machine learning models from stage 1 (Tables SI6 and SI7). This subset of features was then used to train a new RF model.

RF model Set-a performance at Stage 2 reached the accuracy of 92.20%, with the same sensitivity as in stage 1 (86%) but improved specificity (97.41%) and an improved F1 score (0.756). For the RF model using Set-b, this reduced feature set improved test performance, achieving an accuracy of 90.07%, with the same sensitivity (64.00%) but increased specificity (95.69%) and a higher F1 score of 0.696 than in Stage 1.

#### Stage 3: Elbow-based feature optimization

We applied the elbow method (Figs. SI6 Set-a, and SI7 Set-b) to illustrate the relationship between the number of selected features and model classification accuracy. At the plateau, the method achieved a classification accuracy of 95.7% for Set-a and 96.2% for Set-b, suggesting that the top 15 features (in both scenarios) capture the most critical information for sarcopenia classification. As a result, this subset was selected for final model training, balancing high predictive performance with reduced model complexity and enhanced interpretability.

**Fig. 3:**
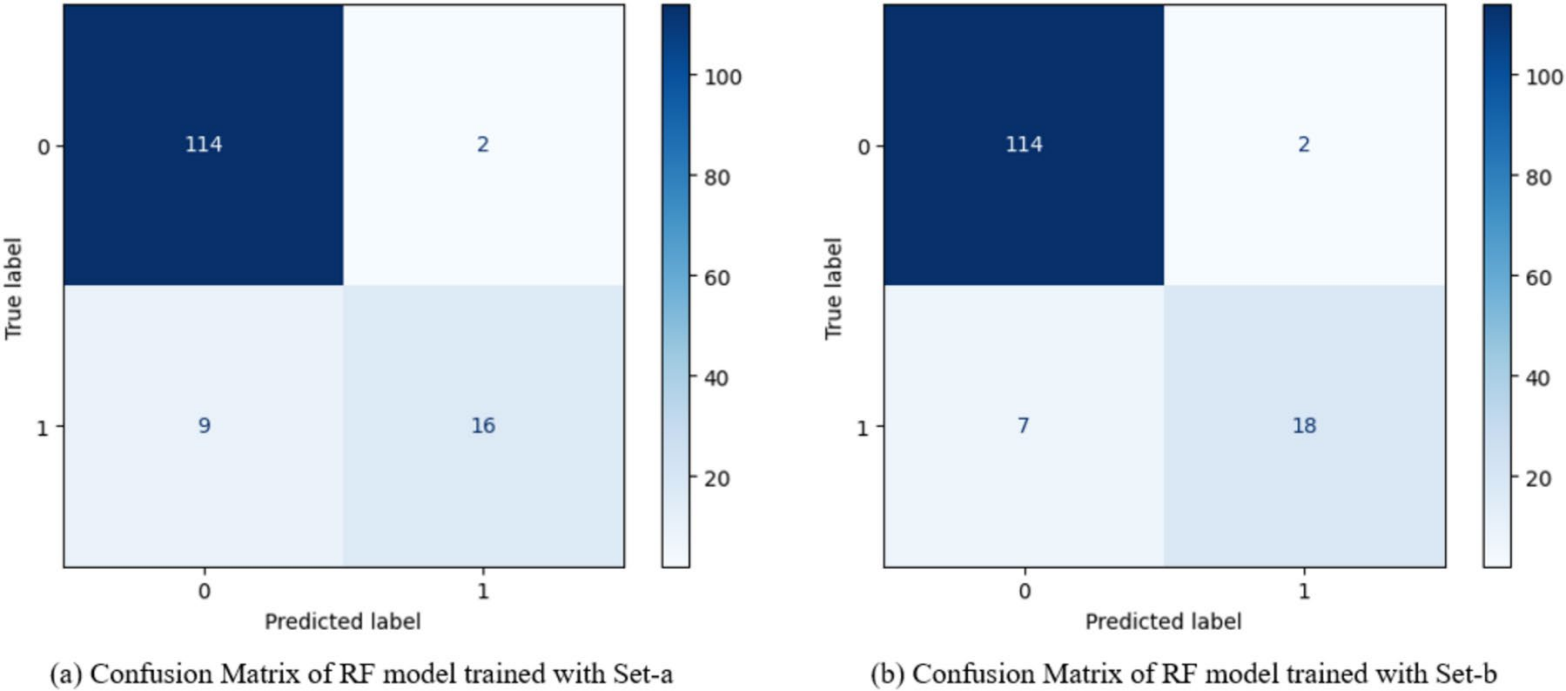
Confusion matrices of prediction models using the **a** Set-a and **b** Set-b data sets with top 15 features

Results from the final RF models trained on the subset of 15 features demonstrated strong classification performance on the testing data set. The Set-a model demonstrated excellent overall accuracy (92.2%) and very high specificity (98.3%), meaning it is highly effective at correctly identifying non-sarcopenic individuals. However, sensitivity (64.0%) is moderate, indicating the model misses some sarcopenia cases (Fig. 3a).

The complementary RF model, Set-b performed very well overall, with a high accuracy of 93.6%, excellent specificity (98.2%), and a notable improvement in sensitivity (72.0%) compared to the previous stage. The F1 score (0.800) indicates a strong balance between precision and recall (Fig. 3b).

#### Statistical comparison of three-stage modelling

The performance of Random Forest models across three stages was evaluated using both AUC and accuracy metrics for Set-a and Set-b. In Set-a, the model achieved an AUC of 0.92 and an accuracy of 91.49% in Stage 1 (all attributes). In Stage 2 (common attributes), the AUC slightly increased to 0.93 with an accuracy of 92.20%. Stage 3 (top 15 attributes) yielded the highest AUC of 0.95 and maintained the same accuracy of 92.20% (Table 1). No statistically significant differences were found between models using DeLong’s test for AUC (Stage 1 vs. 2: *p* = 0.362; Stage 1 vs. 3: *p* = 0.256; Stage 2 vs. 3: *p* = 0.142) or McNemar’s test for accuracy (all *p* = 1.000).

**Table 1:** Classification results of the three-stage RF models

	Accuracy	Sensitivity	Specificity	F1 Score	AUC [95% CI]
RF Set-a—Stage 1	91.49	68.00	96.55	0.739	0.920 [0.841–0.978]
RF Set-a—Stage 2	92.20	68.00	97.41	0.756	0.934 [0.875–0.981]
RF Set-a—Stage 3	92.20	64.00	98.28	0.744	0.945 [0.894–0.984]
RF Set-b—Stage 1	89.36	64.00	94.83	0.681	0.919 [0.845–0.974]
RF Set-b—Stage 2	90.07	64.00	95.69	0.696	0.921 [0.852–0.975]
RF Set-b—Stage 3	93.62	72.00	98.28	0.800	0.951 [0.901–0.989]

In Set-b, Stage 1 showed an AUC of 0.92 and an accuracy of 89.36%. In Stage 2, the AUC remained 0.92 with an accuracy of 90.07%. The model reached its peak performance in Stage 3, with an AUC of 0.95 and an accuracy of 93.62% (Table 1, Fig. SI8). These results indicate that feature refinement led to improved discriminative performance, especially in Stage 3 of both sets. In this model set, DeLong’s test revealed significant improvements in AUC for Stage 3 compared to Stage 1 (*p* = 0.022) and Stage 2 (*p* = 0.009). Accuracy improvements showed a positive trend, though they did not reach statistical significance (Stage 1 vs. 3: *p* = 0.070; Stage 2 vs. 3: *p* = 0.180, McNemar’s test).

### SHAP importance scores for the most influential features

**Fig. 4:**
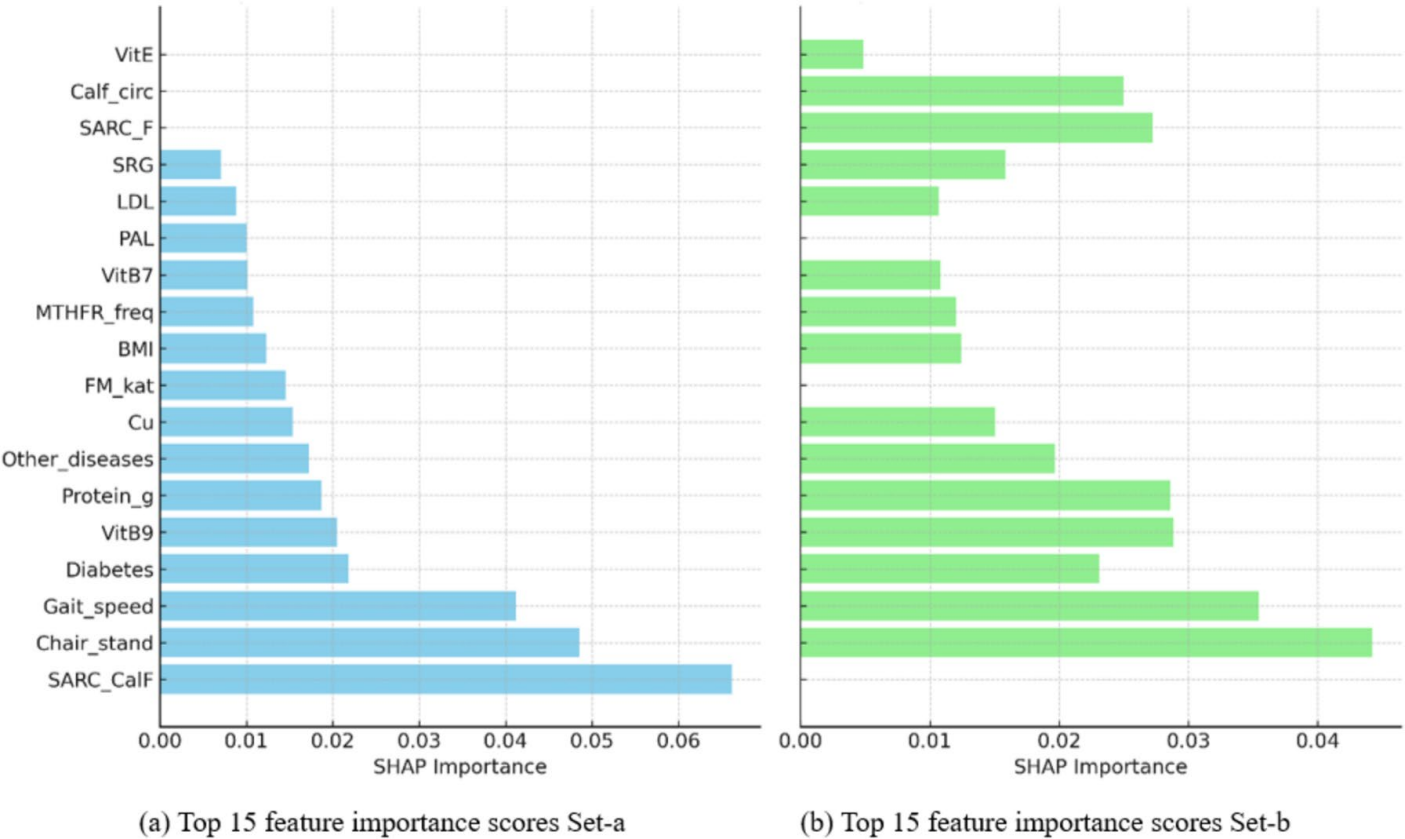
SHAP plots of feature importance scores of the top 15 feature trained with **a** Set-a, and with **b** Set-b for the final RF models

The SHAP importance scores for the most influential features in both models are presented in Fig. 4, and Table 2. In the model with Set-a, SARC–CalF, chair stand, and gait speed were the top predictors, followed by clinical and nutritional factors including diabetes, vitamin B9, protein intake, presence of other diseases, fat mass (FM_kat), copper (Cu), BMI, MTHFR gene frequency, physical activity level (PAL), vitamin B7, LDL, and SRG. Notably, calf circumference was not included in this model, as it is incorporated into the SARC–CalF score. In the model with Set-b, the most important features included chair stand, gait speed, vitamin B9, protein intake, SARC-F, calf circumference, diabetes, other diseases, SRG, Cu, BMI, MTHFR frequency, vitamin B7, LDL, and vitamin E.

**Table 2:** Top 15 SHAP-ranked features and their clinical interpretation

Feature	Set-a (SARC–CalF)	Set-b (SARC-F)	Direction of Influence	Clinical Interpretation	References
Calf Circumference	–	✔	↓ Larger circumference	Simple proxy for muscle mass, used in screening	[21, 22]
Chair Stand Test	✔	✔	↑ Poor performance	Reflects lower body strength and functional decline	[1, 20]
Gait Speed	✔	✔	↑ Slower speed	Slower gait indicates frailty and reduced mobility	[1, 20]
Protein Intake	✔	✔	↓ Higher intake	Supports muscle protein synthesis and mass preservation	[4, 29]
Folate (Vitamin B9)	✔	✔	↓ Higher levels	Important for muscle metabolism and homocysteine regulation	[30, 31]
Diabetes	✔	✔	↑ Presence	Promotes inflammation and muscle wasting	[1, 2]
Other Chronic Diseases	✔	✔	↑ Presence	Comorbidity burden contributes to muscle loss and functional decline	[1, 37]
Copper (Cu)	✔	✔	↓ Higher levels	Trace element important in mitochondrial function and redox balance	[27]
BMI	✔	✔	↑ Low BMI	Low BMI may reflect malnutrition or underdiagnosed sarcopenia	[1, 26]
MTHFR Polymorphism	✔	✔	↑ Risk variant	Impairs folate metabolism, linked to poor muscle outcomes	[32–34]
SRG (Risk Genotype)	✔	✔	↑ Risk genotype	Aggregates sarcopenia-related SNPs; reflects genetic susceptibility	[5, 14]
Vitamin B7 (Biotin)	✔	✔	↓ Higher levels	Supports enzymatic processes involved in muscle function	[27]
LDL	✔	✔	↑ Higher levels	May reflect metabolic and inflammatory dysregulation	[36]
Fat Mass (FM_kat)	✔	–	↑ Higher fat mass	Associated with sarcopenic obesity and reduced muscle quality	[12]
Physical Activity Level	✔	–	↓ Higher level	Preserves strength and delays muscle loss	[6, 11]
Vitamin E	–	✔	↓ Higher levels	Antioxidant properties may protect against oxidative muscle damage	[27]

**Note:**

✔ = Feature included in top 15 of respective model

↑ = Increases sarcopenia risk

↓ = Protective or associated with lower risk

– = Not included (e.g., calf circumference embedded in SARC–CalF)

While both models share a core set of key predictors: chair stand, gait speed, protein intake, vitamin B9, presence of other diseases, diabetes, Cu, BMI, MTHFR gene, vitamin B7, LDL, and SRG, the distinct inclusion of either SARC–CalF or SARC-F plus calf circumference led to subtle differences in feature prioritization.

## Discussion

In this study of 484 older adults, 18% met the diagnostic criteria for sarcopenia according to EWGSOP2 guidelines. By utilizing multimodal data, including physical performance metrics, body composition, nutritional intake, clinical diagnoses, and genetic information, we applied ML models to identify the most relevant risk factors for sarcopenia.

Applying a structured machine learning pipeline to multimodal data, this study compared Random Forest models predicting sarcopenia based on either SARC–CalF (Set-a) or SARC-F (Set-b). The SARC-F-based model (Set-b), optimized using SHAP, ultimately achieved superior predictive performance (AUC = 0.95, Accuracy = 93.62%). While SARC–CalF was highly influential when included (Set-a), key functional measures such as chair stand and gait speed were consistently strong predictors across both models. Notably, SARC-F proved highly effective in Set-b when synergistically combined with multimodal inputs, including an independent contribution from calf circumference. Both models underscored the importance of nutritional factors (e.g., protein and folate) and clinical status (e.g., diabetes and multimorbidity). Despite differences driven by the screening tool, the shared core predictors affirm the reliability of functional, clinical, and nutritional markers in sarcopenia risk assessment, enhanced here through interpretable machine learning. These findings, particularly the high predictive importance of functional measures, align well with established clinical perspectives like the Global Leadership Initiative on Sarcopenia (GLIS) framework, which emphasizes muscle strength, muscle mass, and muscle-specific strength as core components of sarcopenia, while physical performance measures such as gait speed are viewed as clinical outcomes [7]. In our cohort, a significant proportion of participants met criteria for severe sarcopenia, likely accounting for the high predictive importance of gait speed and chair stand across models. These measures capture not only the early decline in muscle strength but also the broader impact of sarcopenia on daily function and independence.

Chair stand and gait speed are widely accepted in both clinical practice and research as indicators of lower body strength and mobility impairment [1]. Prior studies have linked slower gait speed and difficulty with chair rise to disability in activities of daily living (ADLs), reduced quality of life, and increased healthcare costs in older adults across residential, assisted living, and community-based settings [20]. Calf circumference, used in SARC–CalF and as a standalone feature in Set-b, remains a valuable proxy for muscle mass and is recommended for sarcopenia screening due to its strong diagnostic performance and low measurement bias [21]. This reinforces the complementary strengths of both tools and sets the stage for a deeper comparison of their performance within machine learning frameworks.

While SARC–CalF has consistently demonstrated higher sensitivity in traditional screening contexts, largely due to its inclusion of calf circumference [22–24], our findings reveal a more nuanced perspective when machine learning is applied. Surprisingly, the SARC-F-based model (Set-b) outperformed the SARC–CalF-based model (Set-a) in both AUC and accuracy. This performance advantage is likely driven by the synergistic effects of integrating SARC-F with multimodal data, which together enhance overall prediction capacity.

Comparable findings have been reported by Castillo-Olea et al. [25] and Zupo et al. [10], who also identified the predictive value of combining functional status, comorbidities, and nutritional biomarkers in ML-based sarcopenia models. Similarly, Ozgur et al. [26] emphasized handgrip strength, sex, BMI, calf circumference, SARC-F, and comorbidity status as significant predictors. Our results complement these findings and further extend the risk factor profile by incorporating biochemical indicators and genetic susceptibility. Traditional risk marker, like BMI appeared with moderate predictive importance in our models, further reinforcing its continued relevance in evaluating sarcopenia risk.

Other studies showed that vitamin and mineral deficiencies, especially folate, vitamin D, and trace elements, play critical roles in sarcopenia progression, findings echoed in our model's prioritization of folate and copper [27, 28]. Kang et al. (2019), who employed ML algorithms to explore sarcopenia-related risk factors similarly emphasized the importance of nutritional intake, particularly dietary protein in older adults. Moreover, a growing body of evidence, including recent meta-analyses [29], supports the association between inadequate protein intake and increased risk of sarcopenia, with current dietary recommendations suggesting 1.0–1.2 g/kg/day for older adults to preserve lean mass and prevent muscle decline [4].

Folate deficiency has been strongly associated with reduced muscle strength and endurance [30, 31], while polymorphisms in the MTHFR gene may impair folate metabolism and contribute to sarcopenia-related phenotypes [32–34]. Elevated plasma homocysteine, a consequence of impaired folate metabolism, has been linked to reduced walking speed, grip strength, and overall physical performance [35], all consistent with the observed predictive roles of folate and MTHFR frequency in our models.

In addition, LDL was identified as a moderately important predictor of sarcopenia in our models. This aligns with recent findings by Jiang et al. [36], who reported a causal association between elevated LDL levels and increased sarcopenia risk. The link may reflect underlying inflammatory and metabolic pathways, supporting LDL as a modifiable biomarker for early detection and prevention.

### Clinical relevance and application

Importantly, the problem of underdiagnosed sarcopenia has been a common concern across multiple studies [37]. As noted by the International Working Group on Sarcopenia, a large proportion of older adults with functional limitations remain undiagnosed when only classical markers are used [38].

From a clinical standpoint, the best-performing model (Set-b, Stage 3) offers a compelling case for integration into routine workflows for sarcopenia screening and early risk stratification. This Random Forest model, which retains SARC-F, demonstrated statistically significant improvements in AUC compared to earlier stages and achieved the highest overall predictive performance. Importantly, it relies on a concise and interpretable set of 15 features, including objective functional assessments (chair stand, gait speed, calf circumference), nutritional markers (protein and folate intake), clinical indicators (comorbidities, diabetes, BMI, LDL), and genetic markers along with the self-reported functional limitations captured by the SARC-F questionnaire. These variables are readily obtainable in outpatient, primary care, and geriatric assessment settings, without the need for advanced imaging or expensive diagnostics. Given that sarcopenia is often underdiagnosed until functional impairments become evident [1], the model’s balance of high sensitivity and specificity makes it especially suitable for preventive care and early intervention. By combining clinical relevance with practical feasibility, this multimodal screening approach has the potential to enhance detection and inform targeted management strategies in both clinical and community settings.

The comparative SHAP analysis shows that while SARC–CalF offers strong standalone value, SARC-F’s integration into a broader ML framework enhances its predictive capacity. The lower number of false negatives in Set-b suggests that this model is better at detecting sarcopenia cases without sacrificing much in terms of specificity or overall accuracy. This makes it a more favourable option if the clinical goal is to enhance early detection of sarcopenia while still minimizing false positives.

In evaluating clinical applicability, this finding highlights the importance of context in selecting screening strategies. Based on the performance of our machine learning models and the clinical characteristics of the screening tools assessed, we developed a sarcopenia screening selection guide tailored to various healthcare settings (Table 3). This guide aims to help clinicians choose between the SARC-F and SARC–CalF-based approaches depending on available resources, clinical goals, and patient profiles. In settings where rapid, low-resource screening is essential, such as primary care or community outreach, SARC–CalF remains a strong standalone tool due to its simplicity and superior sensitivity reported in prior literature. However, in more advanced settings with access to multimodal patient data, our findings suggest that an ML-enhanced model incorporating SARC-F, along with objective physical performance measures and nutritional indicators, can provide superior diagnostic accuracy and sensitivity. The guide outlines use-case scenarios from low-complexity standalone tools to high-complexity, data-driven models suitable for integration into electronic health records or comprehensive geriatric assessments. This framework is intended to support personalized and context-appropriate screening, enabling earlier detection and more effective intervention for sarcopenia.

**Table 3:** Sarcopenia screening selection guide

Case/scenario	Recommended tool/model	Model type	Complexity	Performance (AUC/accuracy)	Clinical context
Rapid screening in primary care or limited-resource settings	SARC–CalF	Standalone screening tool	Low	High sensitivity (literature)	Easy to implement; no computation required
Basic screening when only SARC-F is available	SARC-F	Single-variable screen	Low	Low	May miss early cases; use only when no other data is available
Clinical assessments with basic physical and nutritional data	Set-b (SARC-F + multimodal)	Traditional composite model	Medium	Moderate	Paper-based risk assessments or clinical checklists
Advanced clinical settings or digital health platforms with comprehensive data access	Set-b (ML model with SARC-F)	Machine Learning (Random Forest)	High	AUC: 0.874/accuracy: 93.6%	Best-performing model in this study; suitable for EHR tools
Geriatric screening with anthropometrics but minimal lab data	Set-a (ML model with SARC–CalF)	Machine Learning (Random Forest)	High	AUC: 0.838/accuracy: 92.2%	Performs well with physical indicators; calf-focused approach

Set-b (SARC-F + multimodal) refers to SARC-F combined with other relevant predictors (e.g., gait speed, chair stand, protein intake, calf circumference, diabetes, fat mass, LDL) in a clinical or statistical model; Set-b (ML model with SARC-F) refers model (Random Forest) that includes SARC-F with all 15 features; Set-a (ML model with SARC–CalF) refers to (Random Forest) that includes SARC–CalF with all 15 features. Clinicians should choose the model based on data availability, workflow complexity, and preference for sensitivity vs. specificity

A significant limitation of this study is the absence of external validation. As an alternative approach, we employed a rigorous internal validation strategy. Specifically, we split our data set into training and testing sets using a 70:30 ratio. During model development, we performed cross-validation on the training set to fine-tune the hyperparameters and reduce the risk of overfitting. The testing set completely held out during training was then used for final model evaluation to assess performance and generalizability.

Although internal validation was performed, the model’s applicability to broader populations remains uncertain without confirmation on independent data sets. External validation will be required in future studies of external data sets and different attributes to confirm the ability to accurately predict sarcopenia and demonstrate utility for screening purposes in various settings or clinical contexts.

## Conclusion

This study demonstrates the value of applying machine learning techniques to multimodal data for the early detection of sarcopenia in older adults. By comparing models incorporating either the SARC-F or SARC–CalF screening tools, we found that both approaches provide clinically relevant insights, but their performance varies depending on feature integration and context. Notably, the SARC-F-based model, when combined with functional, nutritional, clinical, and genetic variables, outperformed the SARC–CalF-based model in terms of overall accuracy and AUC, suggesting that even a simpler screening tool can yield robust predictions when supported by complementary data.

Our findings highlight the importance of functional measures, such as chair stand, gait speed, and calf circumference as well as nutritional and clinical factors, including protein intake, folate levels, and chronic disease status, in predicting sarcopenia. The integration of interpretable machine learning with clinically accessible variables offers a promising direction for enhancing early detection, especially in settings, where advanced diagnostics may not be available.

Finally, we propose a screening selection framework to help clinicians tailor sarcopenia assessments to specific care environments, balancing diagnostic performance, data availability, and complexity.

However, a key limitation of this study is the lack of external validation, which restricts confidence in the generalizability of these findings. To move toward clinical implementation, future work should aim to externally validate these models using independent and larger data sets, as well as assess their longitudinal predictive value in tracking sarcopenia onset or progression over time.

## Supplementary Information

Below is the link to the electronic supplementary material.

Supplementary file1 (DOCX 332 KB)

## Data Availability

The data that support the findings of this study are available from the corresponding author upon reasonable request.
