# Supplementary material for: Identifying risk factors for sarcopenia using machine learning: insights from multimodal data
Source: Eur Geriatr Med. 2025 Jun 5;16(5):1777–88. doi: 10.1007/s41999-025-01245-5 (PMC12528252; doi:10.1007/s41999-025-01245-5)
Supplement: Supplementary file 1 — Supplementary file1 (DOCX 332 KB) [file 41999_2025_1245_MOESM1_ESM.docx]

**Supplementary Information (SI)**

**Identifying Risk Factors for Sarcopenia Using Machine Learning: Insights from Multimodal Data**

Felicita Urzi ^(1,2)^, Domen Šoberl ^(1)^, Ornella Caputo ^(2)^, Marco Narici ^(2)^

1. University of Primorska, Faculty of Mathematics, Natural Sciences and Information Technologies, Koper, Slovenia
2. University of Padova, Department of Biomedical Sciences, Padova, Italy

e-mail address of the corresponding authors: [felicita.urzi@upr.si](mailto:felicita.urzi@upr.si); [marco.narici@unipd.it](mailto:marco.narici@unipd.it)

**Text SI1** Detailed descriptions of diagnostic criteria used for sarcopenia diagnosis.

**Text SI2** Detailed descriptions of SARC-F and SARC-CalF assessments.

**Text SI3** Dietary Intake and Malnutrition Assessment.

**Text SI4** Detailed descriptions of genetic data.

**Table SI1** Description of features included in the ML models.

**Table SI2** Characteristic and proportion of study participants in relation to the living environment.

**Fig. SI1** Diagram showing the separation of features in the machine learning pipelines.

**Fig. SI2** Distribution of diseases in Group 1 (nursing home residents) and Group 2 (independent living older adults).

**Fig. SI3** Distribution of the number of diseases/subject in Group 1 (nursing home residents) and Group 2 (independent living older adults).

**Table SI3** Comparison of baseline characteristics between normal and sarcopenic participants.

**Fig. SI4** Distribution of the number of diseases/subject in non-sarcopenia and sarcopenia group.

**Fig. SI5** Distribution of diseases in non-sarcopenia and sarcopenia group.

**Table SI4** Performance metrics of five ML models for Set-a, models built on the full set of features.

**Table SI5** Performance metrics of five ML models for Set-b, models built on the full set of features.

**Table SI6** Score of features across prediction models for Set-a.

**Table SI7** Score of features across prediction models for Set-b.

**Fig. SI6** Elbow plot showing classification accuracy as a function of the number of top-ranked features for Set-a.

**Fig. SI7** Elbow plot showing classification accuracy as a function of the number of top-ranked features for Set-b.

**Fig. SI8** ROC curves of Random forest models accros three stages for Set-a (Random Forest, excluding SARC-F), and Set-b (Random Forest, excluding SARC-CalF).

**Text SI1 Detailed descriptions of diagnostic criteria used for sarcopenia diagnosis**

**Sarcopenia was assessed according to EWGSOP2 diagnostic criteria** [1] **in a cross-sectional studies.**

*1. Low muscle strength*

Low muscle strength was classified as a hand grip strength <27 kg for men and <16 kg for women. Strength was measured as grip strength using a Handgrip Dynamometer (Jamar Hydraulic Hand Dynamometer, Homecraft Ltd., Nottinghamshire, United Kingdom) and was defined as the best performance of two attempts by the dominant hand.

*2. Low muscle quantity or quality*

Low muscle mass was classified as a skeletal muscle index of less than 8.87 and 6.42 kg/m^2^ for men and women, respectively. Body composition was measured using bioelectrical impedance analysis (BIA; Maltron Bioscan 920, Rayleigh, United Kingdom, 50 kHz). The total skeletal muscle mass of the body was calculated using the equation: skeletal muscle mass (kg) = {(body height^2^/BIA resistance x 0.401) + (sex x 3.825) + [age x (–0.071)]} + 5.102 [2]. Skeletal muscle index (SMI) was calculated as muscle mass (kg) divided by height squared (m^2^).

*3. Low physical performance*

The cut-off points of gait speed <0.8 m/s indicates participants with low physical performance. Physical performance was examined by usual gait speed over a 4 m course. Time was measured (s).

*4. Operational definition of sarcopenia used for sarcopenia diagnosis:*

- Probable sarcopenia is identified by Criterion 1.
- Diagnosis is confirmed by additional documentation of Criterion 2.
- If Criteria 1, 2 and 3 are all met, sarcopenia is considered severe.

We classified the participants as sarcopenic if the criterion 1. and 2. were present (*n* = 11) and as severe sarcopenic if the criterion 1., 2. and 3. were present (*n* = 75). We merged them in one sarcopenic group.

**Text SI2** **Detailed descriptions of SARC-F and SARC-CalF assessments**

*SARC-F test*

The original version of the SARC-F questionnaire [3] was translated into Slovenian and then back-translated into English by a registered English translator and verified by the authors to maintain the conceptual equivalence of the terms used. The SARC-F questionnaire was assessed face to face by investigators. Participant strength, assistance in walking, rising from a chair, stair climbs, and falls were evaluated with answers to the SARC-F questions. To each answer is assigned 0 to 2 points according to the reported difficulty in performing the task in question (0 = no difficulty, 1 = some, and 2 = a lot or unable to do activity). The sum of components serves as a score and ranges from 0 to 10 (0 = best to 10 = worst). Participants defined as having sarcopenia have a total SARC-F score of ≥4.

*SARC-CalF test*

The SARC-CalF test [4] includes the combined value of SARC-F questionnaire components and calf circumference measurements. Calf circumference is measured through scoring: zero representing the absence of low muscle mass (>34 cm for men and >33 cm for women) and 10 for presence (≤34 cm for men and ≤33 cm for women). The sum of components serves as a score and ranges from 0 to 20 (0 = best to 20 = worst). Participants defined as having sarcopenia have a total SARC-CalF score of ≥11 [5].

**Text SI3** **Dietary Intake and Malnutrition Assessment**

Habitual dietary intake was assessed with the 3-day weighed dietary record over two weekdays and on one weekend day. The weight of food intake in grams was converted into energy, macronutrient and micronutrient amounts using the online OPEN (Open Platform for Clinical Nutrition dietary assessment tool (http://www.opkp.si)).

The mini nutritional assessment (MNA - Full Form) was used to assess the nutritional status of the older adults. The MNA questionnaire was assessed face to face by investigators. Malnutrition indication scores ranged from 17 to 30 points. Participants were classified according to achieved scores as follows: from 24 to 30 points (normal nutritional status), from 17 to 23.5 points (at risk of malnutrition), and less than 17 points (malnourished) [6].

**Text SI4** **Detailed descriptions of genetic data**

*Sample Collection and Genotyping*

Saliva samples were collected in the DNA stabilization buffer. The genomic DNA was extracted from saliva using the DNA| OG-500 Kit (DNA Genotek Inc.) following the manufacturer protocol. The DNA quality was evaluated by NanoDrop spectrometry and quantified with the Qubit dsDNA Broad Range Assay Kit (Invitrogen, Q32853). All DNA samples were stored at -80ºC. Polymorphism of the MTHFR 1.p36.2 A > C (rs1801131), ACTN3 11.q13.2 C > T (rs1815739), NRF2 15q21.2 C > A, (rs12594956), VDR FokI 12q13.1 T > C (rs2228570), ADRB2, 5q32 G > A (rs1042713), CX3CR1 3.p22 C > T (rs3732379), and NPAS4 11q13.2 A > G (rs7947391) genes were genotyped using KASP assay based on competitive allele-specific PCR (polymerase chain reaction), according to the manufacturer protocol (LGC, Biosearch Technologies, United Kingdom). The PCR reaction was performed with a final volume of 10 mL per reaction [5 mL genomic DNA (10 ng), 5 mL KASP Master mix (2x), 0.14 mL KASP assay mix]. The protocol was performed with the LightCycler 96 Real-Time PCR System (Roche Molecular Systems, Inc., Pleasanton, CA, United States). Polymorphisms of the candidate genes were genotyped using a standardized genotyping technique (KASP assay), based on competitive allele-specific PCR. The KASP assay exhibited a 93.5% amplification rate and allele call quality of 98%. For samples with missing genotype results, the DNA extraction and genotyping were repeated. All analyses were performed using the negative control. We repeated the genotyping in a randomly chosen sample (10%), using the same technique to ensure genotyping accuracy.

*Calculation of Total Sarcopenia Genetic Risk Score*

Only the gene variants associated with sarcopenia were included in the calculation of the total sarcopenia genetic risk score. To quantify the combined contribution of polymorphisms associated with sarcopenia, we used an algorithm resulting from the accumulation of genotype scores for each individual (Williams and Folland, 2008). The polygenic profile was calculated assuming an additive effect, with all three gene variants given equal weight in the total score. Specifically, each polymorphism genotype was evaluated with a genetic score based on the association with an unfavorable phenotype (i.e., sarcopenia status). The “unfavorable” homozygous genotype was rated 2, the heterozygous genotype 1, and the non-risk homozygous genotype 0. The total SGS was then mathematically converted to a scale from 0 to 100% (where an SGS of 100% represents a “perfect” polygenic profile for sarcopenia, and an SGS of 0% represents the “worst” profile for sarcopenia). Considering the genotype scores of three genes, the total sarcopenia genetic risk scores were calculated according to the equation: SGS = (100/6) x (genotype scores MTHFR C + genotype scores ACTN3 C + genotype scores NRF2).

**Reference**

[1] Cruz-Jentoft AJ, Bahat G, Bauer J, Boirie Y, Bruyère O, Cederholm T, et al. Sarcopenia: revised European consensus on definition and diagnosis. Age Ageing 2019;48:16–31. https://doi.org/10.1093/ageing/afy169.

[2] Janssen I, Heymsfield SB, Baumgartner RN, Ross R. Estimation of skeletal muscle mass by bioelectrical impedance analysis. J Appl Physiol (1985) 2000;89:465–71. https://doi.org/10.1152/jappl.2000.89.2.465.

[3] Malmstrom TK, Morley JE. SARC-F: a simple questionnaire to rapidly diagnose sarcopenia. J Am Med Dir Assoc 2013;14:531–2. https://doi.org/10.1016/j.jamda.2013.05.018.

[4] Barbosa-Silva TG, Menezes AMB, Bielemann RM, Malmstrom TK, Gonzalez MC, Grupo de Estudos em Composição Corporal e Nutrição (COCONUT). Enhancing SARC-F: Improving Sarcopenia Screening in the Clinical Practice. J Am Med Dir Assoc 2016;17:1136–41. https://doi.org/10.1016/j.jamda.2016.08.004.

[5] Urzi F, Šimunič B, Buzan E. Basis for Sarcopenia Screening With the SARC-CalF in Nursing Homes. J Am Med Dir Assoc 2017;18:991.e5-991.e10. https://doi.org/10.1016/j.jamda.2017.07.011.

[6] Vellas B, Guigoz Y, Garry PJ, Nourhashemi F, Bennahum D, Lauque S, et al. The Mini Nutritional Assessment (MNA) and its use in grading the nutritional state of elderly patients. Nutrition 1999;15:116–22. https://doi.org/10.1016/s0899-9007(98)00171-3.

**Table SI1 Description of all features included in the ML models**

| **Category** | **Variables** | **Description** | **Measure/cut-off value** | **Collected data** |
| --- | --- | --- | --- | --- |
| Demographic features | Age | The chronological age of participants. | years | 484 |
|  | Gender | Type of genders. | 1 = male, 2 = female | 484 |
|  |  |  |  |  |
|  | Environment | Living option of participants | 1 = Nursing homes,  2 = Independent living | 484 |
| Sarcopenia questionnaires | SARC-F | 5-item questionnaire. Responses are based on the patient’s perception of his or her limitations in strength, walking ability, rising from a chair, stair climbing and experiences with falls. To each answer is assigned 0 to 2 points according to the reported difficulty in performing the task in question (0 = no difficulty, 1 = some, and 2 = a lot or unable to do activity), with the greatest maximum SARC-F score being 10. | 0 = no sarcopenia (SARC-F score of <4)  1= probable sarcopenia (SARC-F score of ≥4) | 481 |
|  | SARC-CalF | The test includes the combined value of SARC-F questionnaire components (10 points) and calf circumference measurements. Calf circumference is measured through scoring: zero representing the absence of low muscle mass (>34 cm for men and >33 cm for women) and 10 for presence (≤34 cm for men and ≤33 cm for women). The sum of components serves as a score and ranges from 0 to 20 (0 = best to 20 = worst). | 0 = no sarcopenia (SARC-CalF score of <11)  1= probable sarcopenia (SARC-CalF score of ≥11) | 477 |
| Functional parameters | Grip strength | Strength was measured as grip strength with a Handgrip Dynamometer and defined as the best performance of 2 attempts by the dominant hand. | kg  Low muscle strength as hand grip strength <30 kg for men and <20 kg for women. | 484 |
|  | Gait speed | The gait speed was measured by usual gait speed over 4m | m/s  gait speed <0.8 m/s indicate participants with low physical performance. | 484 |
|  | Chair stand test | Chair stand test was measured as time needed to rise five time from the chair. Time was recorded. Based on scaled range the time was converted to points. | Points  0 unable  1 > 16,7s  2 13,7s-16,69s  3 11,2s - 13,69s  4 < 11,19s | 307 |
|  | Calf circumference | Calf_circ was measured using non-elastic tape in sited position. | cm |  |

| **Category** | **Variables** | **Description** | **Measure/cut-off value** | **Collected data** |
| --- | --- | --- | --- | --- |
| Anthropometric features | BMI | Body mass index. Weight (kg) divided by height (m) squared. | kg/m^2^ | 484 |
|  | FM_kat | Fat mass was derived from BIA (bioelectrical impedance analysis) (Maltron Bioscan 920, 50 kHz; Rayleigh, United Kingdom).  1 = lean; 2 = normal; 3 = over weight; 4 = obese | % F:16-27 = 1, 27-32 = 2, 32-37 = 3, >37% =4)  M:10-21 = 1, 21-26 = 2, 26-31 = 3, >31 = 4) | 466 |
|  | TBW | Total body water (TBW) was derived from BIA | % | 466 |
|  | SMI | Total body skeletal muscle mass was calculated  using the BIA equation: skeletal muscle mass (kg) = [(body height^2^/BIA resistance x 0.401) + (sex x 3.825) + (age x (-0.071))] + 5.102. Skeletal muscle index (SMI) was calculated as muscle mass (kg) divided by square of body height (m). | kg/m^2^  Low muscle mass was classified as the skeletal  muscle index less than 8.87 and 6.42 kg/m^2^ in men and women, respectively. | 484 |
|  | CC | Maximal calf circumference (CC) was measured on the right calf with the legs relaxed and feet 20 cm apart from eachother. | cm  >34 cm for men and >33 cm for women | 477 |
|  | Visceral fat | Visceral fat was derived from BIA | point | 172 |
|  | Bone density | Bone density was derived from BIA | kg | 172 |
| Dietary features | Protein | Protein intake (mg) / body weight / day | mg/kg/day | 154 |
|  | Protein | % Energy from Protein | % | 154 |
|  | Carbohydrates | % Energy from Carbohydrates | % | 154 |
|  | Fat | % Energy from Fat | % | 154 |
|  | VitD, | Vitamin D | μg | 154 |
|  | BCAA | Branched-chain amino acid | g | 154 |
|  | LEVC | Leucine | g | 154 |
|  | C:16 | Palmitic acid | g | 154 |
|  | C:18 | Stearic acid | g | 154 |
|  | ω3 | n-3 fatty acid | g | 154 |
|  | ω6 | n-6 fatty acid | g | 154 |
|  | HOL | Cholesterol | mg | 154 |
|  | VitE | Vitamin E | mg | 154 |
|  | VitK | Vitamin K | µg | 154 |
|  | VitC | Vitamin C (mg), | mg | 154 |
|  | VitB1 | Thiamine - vitamin B1 | mg | 154 |
|  | VitB2 | Riboflavin -vitamin B2 | mg | 154 |
|  | VitB6 | Vitamin B6 | mg | 154 |
|  | PAL | PAL was calculated from the total energy expenditure (TEE) and BMR (basal metabolic rate) with the equation PAL = TEE/BMR. The PAL’s were classified according FAO/WHO/UNU expert consultation on human energy requirements (2004). | 1.2 – 1.3 no physical activity  1.40 – 1.69 light activity  1.70 – 1.99 moderately active  2.00 – 2.40 vigorously active | 154 |

| **Category** | **Variables** | **Description** | **Measure/cut-off value** | **Collected data** |
| --- | --- | --- | --- | --- |
|  | PantA | Pantothenic acid – vitamin B5 | mg | 154 |
|  | VitB7 | Biotin - vitamin B7 | µg | 154 |
|  | VitB9 | Folate - vitamin B9 | µg | 154 |
|  | VitB12 | Vitamin B12 | µg | 154 |
|  | K | Potassium | mg | 154 |
|  | Ca | Calcium | mg | 154 |
|  | Mg | Magnesium | mg | 154 |
|  | Fe | Iron | mg | 154 |
|  | Cu | Copper | µg | 154 |
|  | Se | Selenium | µg | 154 |
|  | Zn | Zinc | µg | 154 |
|  | MNA | The mini nutritional assessment (MNA) was used to assess the nutritional status. Participants were classified according to achieved scores. | Points: normal nutritional status: from 24 to 30,  at risk of malnutrition from 17 to 23,  malnourished: less than 17 points | 109 |
| Health features | COPD | Chronic Obstructive Pulmonary Disease | Presence = 1, absence = 0 | 146 |
|  | Diabetes M. | Type 2 diabetes | Presence = 1, absence = 0 | 146 |
|  | Hypertension | Hypertension | Presence = 1, absence = 0 | 146 |
|  | Heart disease | Heart disease | Presence = 1, absence = 0 | 146 |
|  | Depression | Depression | Presence = 1, absence = 0 | 146 |
|  | Other diseases | Number of any additional diseases suffered by individuals | Sum of chronic diseases |  |
|  | Number of diseases | Total number of diseases | Sum of all diseases |  |
|  | MCI | Mild cognitive impairment - reported by a doctor | Presence = 1, absence = 0 | 300 |
| Genetic features | MTHFR_freq  SNP | Methylenetetrahydrofolate reductase  Polymorphism of the MTHFR 1.p36.2 A > C (rs1801131) | genotype scores  0= AA, 1= AC, 2= CC | 189 |
|  | MTHFR_dom | Methylenetetrahydrofolate reductase - SNP | Dominant genotype (AA/AC+CC) | 189 |
|  | MTHFR_rec | Methylenetetrahydrofolate reductase - SNP | Recessive genotype (AA+AC/CC) | 189 |
|  | VDR_freq  SNP | Vitamin D receptor  Polymorphism of the VDR FokI 12q13.1 T > C (rs2228570) | genotype scores  0=TT, 1=CT, 2=CC | 189 |
|  | VDR_dom | Vitamin D receptor - SNP | Dominant genotype (TT/TC+CC) | 189 |
|  | VDR_rec | Vitamin D receptor - SNP | Recessive genotype (TT+TC/CC) | 189 |
|  | ACTN3_freq  SNP | Alpha-actinin-3  Polymorphism of the ACTN3 11.q13.2 C > T (rs1815739) | genotype scores  0=CC, 1=CT, 2=TT | 189 |
|  | ACTN3_dom | Alpha-actinin-3 SNP | Dominant genotype (CC/CT+TT) | 189 |
|  | ACTN3_rec | Alpha-actinin-3 SNP | Recessive genotype (CC+CT/TT) | 189 |

| **Category** | **Variables** | **Description** | **Measure/cut-off value** | **Collected data** |
| --- | --- | --- | --- | --- |
|  | NRF2_freq  SNP | Nuclear respiratory factor 2  Polymorphism of the NRF2 15q21.2 C > A (rs12594956) | genotype scores  0=AA, 1=AC, 2=CC | 189 |
|  | NRF2_dom | Nuclear respiratory factor 2 - SNP | Dominant genotype (AA/AC+CC) | 189 |
|  | NRF2_rec | Nuclear respiratory factor 2 - SNP | Recessive genotype (AA+AC/CC) | 189 |
|  | SRG | Total Sarcopenia Genetic Risk Score  The polygenic profile was calculated assuming an additive effect, with all gene variants given equal weight in the total score. | (%) | 189 |
| Biochemical - Blood test | S-Glucose | Glucose mmol_L | mmol/L | 135 |
|  | S-CRP | High-Sensitivity C-Reactive Protein __mg_L_ | mg/L | 115 |
|  | S-Total cholesterol | S_Holesterol__mmol_L_ | mmol/L | 115 |
|  | S-Triglycerides | Triglycerides __mmol_L_ | mmol/L | 115 |
|  | S-HDL cholesterol | High-Density Lipoprotein Cholesterol mmol_L_ | mmol/L | 115 |
|  | S-LDL cholesterol | Low-Density Lipoprotein Cholesterol mmol_L_ | mmol/L | 115 |

*Note*: the outcome (sarcopenia) was a binary variable, and were coded as 0 (no sarcopenia) and 1 (sarcopenia). Frequency (%); *b*, Wilcoxon rank sum test; Pearson’s Chi-squared test.

**Fig. SI1**


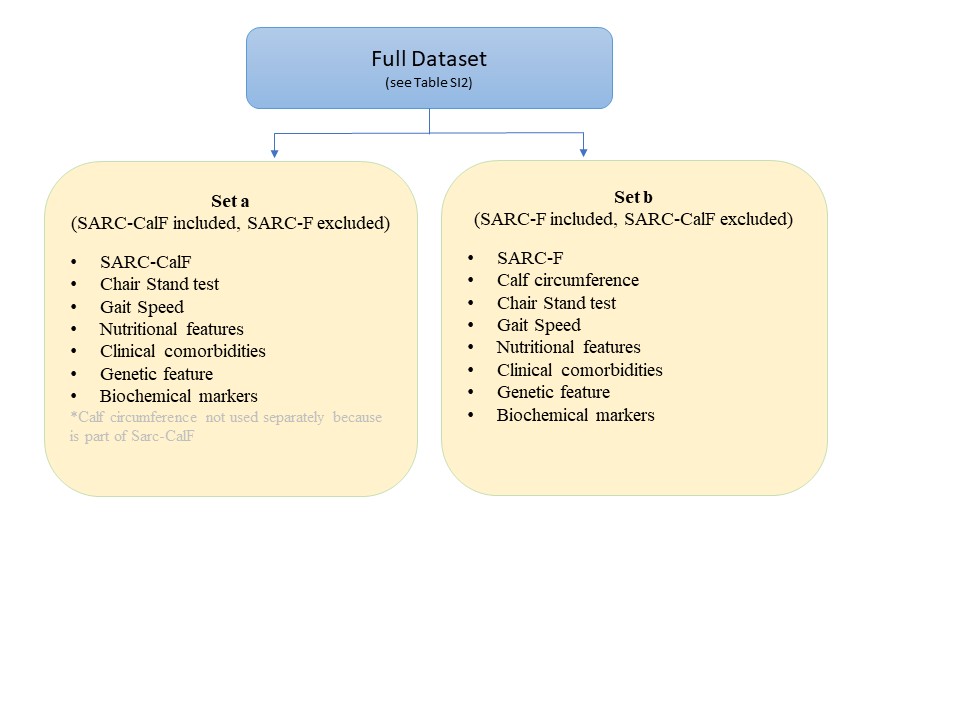


Diagram showing the separation of features in the machine learning pipelines. To avoid collinearity and redundancy from overlapping components between SARC-F and SARC-CalF, two mutually exclusive datasets were constructed. Set-a included SARC-CalF and excluded SARC-F (with calf circumference embedded in the score), while Set-b included SARC-F and excluded SARC-CalF, allowing calf circumference to be considered as an independent variable. Both sets retained the same pool of additional functional, nutritional, clinical, and genetic features.**Table SI2** **Baseline characteristics and proportions of study participants by living environment**

| **Characteristic** | **Total sample**  **(n = 484)** |  | **Independent living**  **(n = 254)** |  | **Nursing homes**  **(n = 230)** |  |
| --- | --- | --- | --- | --- | --- | --- |
|  | **M (SD) / n %)** |  | **M (SD) / n %)** |  | **M (SD) / n %)** | ***P-value*** |
| Age (years) | 76 (9.7) |  | 70 (5.8) |  | 83 (8.2) | <0.001 |
| Gender (men) | 144 (30%) |  | 62 (24%) |  | 82 (35%) | 0.007 |
| EWGSOP2 class |  |  |  |  |  | <0.001 |
| No sarcopenia | 398 (82%) |  | 245 (96%) |  | 153 (67%) |  |
| Sarcopenia | 86 (18%) |  | 9 (4%) |  | 77 (33%) |  |
| SARC-F class |  |  |  |  |  | <0.001 |
| No sarcopenia | 359 (74.6%) |  | 236 (93%) |  | 123 (53%) |  |
| Sarcopenia | 122 (25.4.2%) |  | 18 (7%) |  | 104 (45%) |  |
| SARC-CalF class |  |  |  |  |  | <0.001 |
| No sarcopenia | 388 (81.3%) |  | 235 (93%) |  | 153 (66%) |  |
| Sarcopenia | 89 (18.7%) |  | 15 (6%) |  | 74 (32%) |  |
| Handgrip strength (kg) | 24.1 (9.3) |  | 29 (8.8) |  | 19 (8.4) | <0.001 |
| Skeletal muscle index (kg/m^2^) | 7.4 (1.4) |  | 7.6 (1.2) |  | 7.3 (1.3) | 0.218 |
| Gait speed (m/s) | 0.90 (0.4) |  | 1.2 (0.2) |  | 0.52 (0.2) | <0.001 |
| Calf circumference (cm) | 36 (2.9) |  | 36 (2.8) |  | 35 (3.5) | 0.021 |
| Body mass index (kg/m^2^) | 27.5 (4.9) |  | 26 (4.3) |  | 28 (5.3) | 0.017 |
| Fat mass (%) | 36.1 (7.3) |  | 35.6 (8.5) |  | 36.6 (7.9) | 0.187 |
| Total body water (%) | 47.6 (4.9) |  | 49.1 (5.8) |  | 45.8 (6.2) | <0.001 |

*Note*. Living environment. 1. Nursing homes; 2. Independent living; EWGSOP = European Working Group on Sarcopenia in Older People. Sarcopenia: low muscle strength (men <27 kg; women <16 kg), and low muscle mass, cut off point ≤8.87 kg/m2 (in men) and ≤6.42 kg/m^2^ (in women), and/or low physical performance (gait speed <0.8 m/s).

**Fig. SI2**

Distribution of diseases in Group 1 (nursing home residents) and Group 2 (independent living older adults)

**Fig. SI3**

Distribution of the number of diseases/subjects in Group 1 (nursing home residents) and Group 2 (independent living older adults)

**Table SI3** **Comparison of baseline characteristics between pooled group with sarcopenia and without sarcopenia classified by EWGSOP2**

| **Variables** | **Total sample**  **N = 484**  ***M (SD) / n %)*** | **Non-Sarcopenia**  **N = 397**  ***M (SD) / n %)*** | **Sarcopenia**  **N = 87**  ***M (SD) / n %)*** | ***P-value^a^*** |
| --- | --- | --- | --- | --- |
| SARC-F classification |  | 359 (74.6%) | 122 (25.4%) | <0.001 |
| SARC-CalF class. |  | 388 (81.3%) | 89 (18.7%) | <0.001 |
| Living environment |  |  |  |  |
| Nursing homes | 230 (48%) | 153 (67%) | 77 (33%) |  |
| Independent living | 254 (52%) | 245 (96%) | 9 (4%) |  |
| Age (year) | 75.8 (9.7) | 74.1 (9.2) | 83.5 (8.4) | <0.001 |
| Gender (male) | 144 (30%) | 112 (28%) | 32 (37%) | <0.001 |
| Handgrip strength (kg) | 24.1 (9.3) | 26.2 (8.7) | 14.3 (4.3) | <0.001 |
| men |  | 35.8 (9.3) | 17.6 (4.8) |  |
| women |  | 22.9 (6.9) | 11.6 (3.2) |  |
| SMI (kg/m^2^) | 7.4 (1.4) | 7.6 (1.1) | 6.5 (0.9) | <0.001 |
| men |  | 9.10 (0.9) | 7.53 (0.8) |  |
| women |  | 7.10 (0.6) | 5.92 (0.4) |  |
| Calf circumference (cm) | 35.8 (2.9) | 36.3 (2.7) | 33.1 (2.7) | <0.001 |
| men |  | 36.9 (2.8) | 34.0 (3.3) |  |
| women |  | 36.3 (3.1) | 36.3 (3.1) |  |
| Gait speed (m/s) | 0.90 (0.4) | 0.98 (0.4) | 0.52 (0.2) | <0.001 |
| Chair stand test (cat) | 2.6 (1.2) | 2.8 (1.1) | 2.1 (1.2) | <0.001 |
| Body mass index (kg/m^2^) | 27.5 (4.9) | 28.1 (4.9) | 24.4 (3.7) | <0.001 |
| Fat mass (%) | 36.1 (7.3) | 36.7 (7.2) | 33.5 (7.0) | <0.001 |
| Total Body Water (%) | 47.6 (4.9) | 48.2 (4.3) | 47.5 (5.0) | 0.056 |
| Visceral fat (point) | 13.1 (5.2) | 13.02 (4.9) | 13.3 (6.0) | 0.742 |
| Bone density (kg) | 2.5 (0.5) | 2.5 (0.5) | 2.3 (0.4) | 0.050 |
| COPD (yes) | 7 (4.8%) | 5 (4.4%) | 2 (6.0%) | <0.497 |
| Hypertension (yes) | 68 (46%) | 49 (43%) | 19 (57%) | 0.107 |
| Diabetes M. (yes) | 33 (22%) | 10 (9.1%) | 8 (25%) | 0.031 |
| Heart disease (yes) | 26 (18%) | 17 (15%) | 9 (27%) | 0.090 |
| Depression (yes) | 13 (9%) | 10 (8%) | 3 (9%) | 0.599 |
| Mild cognitive impair (yes) | 60 (20%) | 48 (18%) | 12 (35%) | 0.002 |
| Other diseases (kat) | 146.0 | 69.8 | 86.0 | 0.036 |
| Protein (g / kg BM / day) | 1.0 (0.3) | 1.1 (0.2) | 1.0 (0.3) | 0.036 |
| Protein (%) | 16.0 (2.7) | 16.0 (2.9) | 15.9 (1.6) | 0.886 |
| Carbohydrates (%) | 48 (6.9) | 48 (7.0) | 47 (7.7) | 0.591 |
| Fat (%) | 34 (6.4) | 33 (6.3) | 35 (6.4) | 0.272 |
| Vitamin D (μg) | 2.6 (2.6) | 2.6 (2.6) | 2.5 (2.8) | 0.447 |
| BCAA (g) | 10 (2.7) | 10 (2.6) | 9.8 (3.1) | 0.516 |
| Leucine (g) | 4.1 (1.1) | 4.2 (1.0) | 4.0 (1.2) | 0.459 |
| Palmitic acid (g) | 10.5 (3.1) | 10.5 (3.2) | 10.6 (2.9) | 0.946 |
| Stearic acid (g) | 4.4 (1.5) | 4.5 (1.5) | 4.5 (1.5) | 0.935 |
| n-3 fatty acid (g) | 1.1 (1.0) | 1.2 (1.1) | 0.8 (0.5) | 0.022 |
| n-6 fatty acid (g) | 7.7 (4. 2) | 7.9 (4.3) | 6.8 (3.5) | 0.226 |
| Cholesterol (g) | 207 (91) | 213 (93) | 173 (71) | 0.012 |
| Vitamin E (mg) | 9.9 (3.1) | 10.2 (3.1) | 8.2 (2.3) | 0.003 |
| Vitamin K (μg) | 142 (161) | 155 (173) | 78.3 (39.4) | 0.028 |
| Vitamin C (mg) | 84.4 (46.4) | 88.6 (48.1) | 63.6 (30.3) | 0.014 |
| Vitamin B1 (mg) | 1.0 (0.4) | 1.1 (0.5) | 0.8 (0.3) | 0.030 |
| Vitamin B2 (mg) | 1.3 (0.3) | 1.4 (0.4) | 1.3 (0.4) | 0.142 |
| Vitamin B6 (mg) | 1.4 (0.6) | 1.5 (0.6) | 1.2 (0.3) | 0.030 |
| Vitamin B5 (mg) | 4.3 (1.6) | 4.3 (1.4) | 4.2 (2.3) | 0.119 |
| Vitamin B7 (μg) | 21.6 (11.7) | 22.7 (11.7) | 16.0 (10.1) | 0.009 |
| Vitamin B9 (μg) | 227 (108) | 238 (109) | 172 (84) | 0.005 |
| Vitamin B12 (μg) | 2.8 (1.6) | 2.8 (1.8) | 2.5 (0.5) | 0.366 |
| Potassium (g) | 2661 (727) | 2730 (738) | 2316 (564) | 0.009 |
| Calcium (g) | 783 (259) | 777 (266) | 814 (219) | 0.510 |
| Magnesium (g) | 336 (138) | 348 (146) | 277 (65) | 0.018 |
| Iron (g) | 13.2 (3.7) | 13.4 (3.9) | 12.1 (2.9) | 0.118 |
| Copper (mg) | 841 (842) | 936 (851) | 366 (617) | 0.002 |
| Selenium (μg) | 54.4 (21.5) | 54.9 (20.6) | 51.6 (26.1) | 0.193 |
| Zinc (mg) | 7.8 (1.4) | 7.9 (1.3) | 7.6 (1.6) | 0.547 |
| PAL | 1.4 (0.1) | 1.4 (0.1) | 1.3 (0.1) | 0.014 |
| Glucose | 5.4 (1.4) | 5.4 (1.4) | 4.9 (0.9) | 0.168 |
| CRP | 4.5 (1.5) | 4.6 (1.3) | 4.0 (2.8) | 0.455 |
| Cholesterol | 5.9 (1.1) | 5.8 (1.1) | 6.4 (0.7) | 0.147 |
| Triglycerides | 1.6 (0.6) | 1.2 (0.6) | 1.1 (0.4) | 0.590 |
| HDL | 1.6 (0.4) | 1.6 (0.4) | 1.5 (0.4) | 0.731 |
| LDL | 3.9 (0.9) | 3.8 (0.9) | 4.4 (0.6) | 0.043 |

*a*, Wilcoxon rank sum test; Pearson’s Chi-squared test. SMI, skeletal muscle index; COPD, Chronic obstructive pulmonary disease; BCAA, Branched-Chain Amino Acids.

**Fig. SI4**

Distribution of the number of diseases/subjects in non-sarcopenia and sarcopenia group

**Fig. SI5**

Distribution of diseases in non-sarcopenia and sarcopenia group**Table SI4 Performance metrics of five ML models for Set-a, models built on the full set of features**

| **Classification algorithms** | **CA validation** | **CA test** | **Sensitivity** | **Specificity** | **F1 Score** | **AUC** |
| --- | --- | --- | --- | --- | --- | --- |
| Random forest (RF) | 97.77 | 91.49 | 68.00 | 96.55 | 0.739 | 0.92 |
| Gradient boosting model (GB) | 95.17 | 88.65 | 76.00 | 91.38 | 0.704 | 0.91 |
| Support Vector Machines (SVM) | 92.38 | 87.94 | 72.00 | 91.38 | 0.679 | 0.91 |
| Decision Trees (DT) | 87.75 | 85.11 | 76.00 | 87.07 | 0.644 | 0.82 |
| Neural Networks (NN) | 75.07 | 78.72 | 64.00 | 81.9 | 0.516 | 0.75 |

"CA validation" refers to the classification accuracy obtained using 10-fold cross-validation on the training set, while "CA test" represents the classification accuracy obtained on the separate test set.

## **Table SI5 Performance metrics of five ML models for Set-b, models built on the full set of features**

| **Classification algorithms** | **CA validation** | **CA test** | **Sensitivity** | **Specificity** | **F1 Score** | **AUC** |
| --- | --- | --- | --- | --- | --- | --- |
| Random forest (RF) | 97.40 | 89.36 | 64.00 | 94.83 | 0.681 | 0.92 |
| Gradient boosting model (GB) | 95.92 | 89.36 | 68.00 | 93.97 | 0.694 | 0.93 |
| Support Vector Machines (SVM) | 92.95 | 87.94 | 72.00 | 91.38 | 0.679 | 0.91 |
| Decision Trees (DT) | 91.08 | 85.11 | 68.00 | 88.79 | 0.618 | 0.78 |
| Neural Networks (NN) | 75.28 | 78.72 | 28.00 | 89.66 | 0.318 | 0.75 |

"CA validation" refers to the classification accuracy obtained using 10-fold cross-validation on the training set, while "CA test" represents the classification accuracy obtained on the separate test set.

**Table SI6 Score of features across prediction models for Set-a**

| **ID** | **DT** | **RF** | **SVM** | **GB** | **NN** | **Sum** |
| --- | --- | --- | --- | --- | --- | --- |
|  |  |  |  |  |  |  |
| BMI | 1 | 1 | 1 | 1 | 1 | 5 |
| Ca | 1 | 1 | 1 | 1 | 1 | 5 |
| Chair_stand | 1 | 1 | 1 | 1 | 1 | 5 |
| Cu | 1 | 1 | 1 | 1 | 1 | 5 |
| Depression | 1 | 1 | 1 | 1 | 1 | 5 |
| Fat | 1 | 1 | 1 | 1 | 1 | 5 |
| Fe | 1 | 1 | 1 | 1 | 1 | 5 |
| Mg | 1 | 1 | 1 | 1 | 1 | 5 |
| MNA | 1 | 1 | 1 | 1 | 1 | 5 |
| SARC_CalF | 1 | 1 | 1 | 1 | 1 | 5 |
| VitB9 | 1 | 1 | 1 | 1 | 1 | 5 |
| VitE | 1 | 1 | 1 | 1 | 1 | 5 |
| ADRB2_dom | 1 | 1 | 1 | 1 | 0 | 4 |
| Age | 1 | 1 | 0 | 1 | 1 | 4 |
| Charbohydrates | 1 | 1 | 1 | 1 | 0 | 4 |
| CRP | 1 | 1 | 0 | 1 | 1 | 4 |
| Diabetes | 1 | 1 | 0 | 1 | 1 | 4 |
| FM_kat | 0 | 1 | 1 | 1 | 1 | 4 |
| Gait_speed | 0 | 1 | 1 | 1 | 1 | 4 |
| K | 1 | 0 | 1 | 1 | 1 | 4 |
| LDL | 1 | 1 | 1 | 1 | 0 | 4 |
| MTHFR_dom | 1 | 1 | 1 | 1 | 0 | 4 |
| MTHFR_freq | 0 | 1 | 1 | 1 | 1 | 4 |
| MTHFR_rec | 1 | 1 | 1 | 1 | 0 | 4 |
| n_diseases | 0 | 1 | 1 | 1 | 1 | 4 |
| Other_diseases | 0 | 1 | 1 | 1 | 1 | 4 |
| PantA | 0 | 1 | 1 | 1 | 1 | 4 |
| Protein_g | 1 | 1 | 0 | 1 | 1 | 4 |
| SRG | 1 | 1 | 1 | 0 | 1 | 4 |
| TBW | 1 | 1 | 0 | 1 | 1 | 4 |
| VitB12 | 1 | 1 | 1 | 1 | 0 | 4 |
| VitB7 | 0 | 1 | 1 | 1 | 1 | 4 |
| VitC | 0 | 1 | 1 | 1 | 1 | 4 |
| VitK | 1 | 1 | 0 | 1 | 1 | 4 |
| Zn | 0 | 1 | 1 | 1 | 1 | 4 |
| BCAA | 1 | 0 | 1 | 0 | 1 | 3 |
| C_16 | 1 | 0 | 1 | 1 | 0 | 3 |
| C_18 | 1 | 1 | 1 | 0 | 0 | 3 |
| Cholesterol | 1 | 1 | 0 | 0 | 1 | 3 |
| HDL | 1 | 1 | 0 | 1 | 0 | 3 |
| PAL | 1 | 1 | 0 | 1 | 0 | 3 |
| VDR_rec | 1 | 1 | 1 | 0 | 0 | 3 |
| VitD | 1 | 0 | 1 | 0 | 1 | 3 |
| ω3 | 0 | 1 | 0 | 1 | 1 | 3 |
| ω6 | 0 | 1 | 1 | 0 | 1 | 3 |

| **ID** | **DT** | **RF** | **SVM** | **GB** | **NN** | **Sum** |
| --- | --- | --- | --- | --- | --- | --- |
| ACTN3_freq | 1 | 0 | 0 | 0 | 1 | 2 |
| ADRB2_freq | 1 | 1 | 0 | 0 | 0 | 2 |
| Bone_density | 0 | 1 | 0 | 1 | 0 | 2 |
| Glucose | 1 | 0 | 0 | 1 | 0 | 2 |
| Hypertension | 1 | 1 | 0 | 0 | 0 | 2 |
| mild_cogitive_impair | 0 | 0 | 0 | 1 | 1 | 2 |
| LEVC | 0 | 1 | 0 | 1 | 0 | 2 |
| NRF2_dom | 1 | 0 | 1 | 0 | 0 | 2 |
| NRF2_rec | 1 | 0 | 0 | 0 | 1 | 2 |
| Se | 1 | 0 | 0 | 0 | 1 | 2 |
| Visceral_fat | 0 | 0 | 1 | 0 | 1 | 2 |
| VitB2 | 0 | 1 | 0 | 1 | 0 | 2 |
| ADRB2_rec | 0 | 0 | 0 | 1 | 0 | 1 |
| Heart_disease | 0 | 0 | 0 | 1 | 0 | 1 |
| NRF2_freq | 0 | 1 | 0 | 0 | 0 | 1 |
| Triglycerides | 0 | 1 | 0 | 0 | 0 | 1 |
| VDR_freq | 1 | 0 | 0 | 0 | 0 | 1 |
| VDR_dom | 0 | 0 | 1 | 0 | 0 | 1 |
| VitB6 | 0 | 1 | 0 | 0 | 0 | 1 |

## **Table SI7 Score of features across prediction models for Set-b**

| **ID** | **DT** | **RF** | **SVM** | **GB** | **NN** | **Sum** |
| --- | --- | --- | --- | --- | --- | --- |
| Age | 1 | 1 | 1 | 1 | 1 | 5 |
| BMI | 1 | 1 | 1 | 1 | 1 | 5 |
| Ca | 1 | 1 | 1 | 1 | 1 | 5 |
| Calf_circ | 1 | 1 | 1 | 1 | 1 | 5 |
| Chair_stand | 1 | 1 | 1 | 1 | 1 | 5 |
| Cu | 1 | 1 | 1 | 1 | 1 | 5 |
| Diabetes | 1 | 1 | 1 | 1 | 1 | 5 |
| MNA | 1 | 1 | 1 | 1 | 1 | 5 |
| SARC_F | 1 | 1 | 1 | 1 | 1 | 5 |
| VitE | 1 | 1 | 1 | 1 | 1 | 5 |
| Zn | 1 | 1 | 1 | 1 | 1 | 5 |
| C_18 | 1 | 1 | 1 | 1 | 0 | 4 |
| Charbohydrates | 0 | 1 | 1 | 1 | 1 | 4 |
| Gait_speed | 1 | 1 | 1 | 1 | 0 | 4 |
| Fat | 0 | 1 | 1 | 1 | 1 | 4 |
| K | 1 | 1 | 1 | 0 | 1 | 4 |
| MTHFR_freq | 0 | 1 | 1 | 1 | 1 | 4 |
| n_diseases | 1 | 1 | 1 | 1 | 0 | 4 |
| Other_diseases | 0 | 1 | 1 | 1 | 1 | 4 |
| Protein_g | 1 | 1 | 0 | 1 | 1 | 4 |
| SRG | 1 | 1 | 1 | 0 | 1 | 4 |
| TBW | 1 | 1 | 0 | 1 | 1 | 4 |
| VitC | 0 | 1 | 1 | 1 | 1 | 4 |
| VitD | 0 | 1 | 1 | 1 | 1 | 4 |
| VitK | 0 | 1 | 1 | 1 | 1 | 4 |
| ω6 | 0 | 1 | 1 | 1 | 1 | 4 |
| ADRB2_freq | 1 | 1 | 0 | 1 | 0 | 3 |
| ADRB2_rec | 1 | 1 | 0 | 1 | 0 | 3 |
| BCAA | 0 | 1 | 1 | 0 | 1 | 3 |
| C_16 | 1 | 0 | 1 | 0 | 1 | 3 |
| Cholesterol | 0 | 1 | 1 | 0 | 1 | 3 |
| Fe | 0 | 0 | 1 | 1 | 1 | 3 |
| LDL | 1 | 1 | 0 | 1 | 0 | 3 |
| Mg | 0 | 1 | 1 | 0 | 1 | 3 |
| MTHFR_rec | 0 | 1 | 1 | 1 | 0 | 3 |
| PAL | 1 | 1 | 0 | 1 | 0 | 3 |
| PantA | 1 | 1 | 0 | 1 | 0 | 3 |
| Triglycerides | 1 | 1 | 0 | 1 | 0 | 3 |
| VDR_dom | 1 | 0 | 1 | 1 | 0 | 3 |
| VDR_rec | 1 | 1 | 1 | 0 | 0 | 3 |
| VDR_freq | 1 | 1 | 0 | 1 | 0 | 3 |
| Visceral_fat | 0 | 0 | 1 | 1 | 1 | 3 |
| VitB2 | 1 | 1 | 0 | 1 | 0 | 3 |
| VitB7 | 1 | 1 | 0 | 0 | 1 | 3 |
| VitB9 | 0 | 1 | 1 | 0 | 1 | 3 |
| ω3 | 1 | 1 | 0 | 1 | 0 | 3 |
| **ID** | **DT** | **RF** | **SVM** | **GB** | **NN** | **Sum** |

| Bone_density | 0 | 1 | 1 | 0 | 0 | 2 |
| --- | --- | --- | --- | --- | --- | --- |
| CRP | 0 | 1 | 0 | 0 | 1 | 2 |
| Depression | 1 | 0 | 0 | 1 | 0 | 2 |
| FM_kat | 0 | 1 | 0 | 1 | 0 | 2 |
| Glucose | 1 | 1 | 0 | 0 | 0 | 2 |
| Hypertension | 1 | 0 | 1 | 0 | 0 | 2 |
| LEVC | 0 | 0 | 0 | 1 | 1 | 2 |
| MTHFR_dom | 0 | 1 | 0 | 1 | 0 | 2 |
| NRF2_rec | 1 | 0 | 0 | 0 | 1 | 2 |
| Se | 0 | 1 | 0 | 0 | 1 | 2 |
| VitB6 | 1 | 0 | 0 | 1 | 0 | 2 |
| VitB12 | 0 | 1 | 0 | 1 | 0 | 2 |
| ACTN3_freq | 1 | 0 | 0 | 0 | 0 | 1 |
| ADRB2_dom | 0 | 0 | 0 | 1 | 0 | 1 |
| Heart_disease | 0 | 0 | 1 | 0 | 0 | 1 |
| HDL | 0 | 1 | 0 | 0 | 0 | 1 |
| mild_cogitive_impair | 0 | 0 | 0 | 0 | 1 | 1 |
| NRF2_freq | 1 | 0 | 0 | 0 | 0 | 1 |

**Fig. SI6**


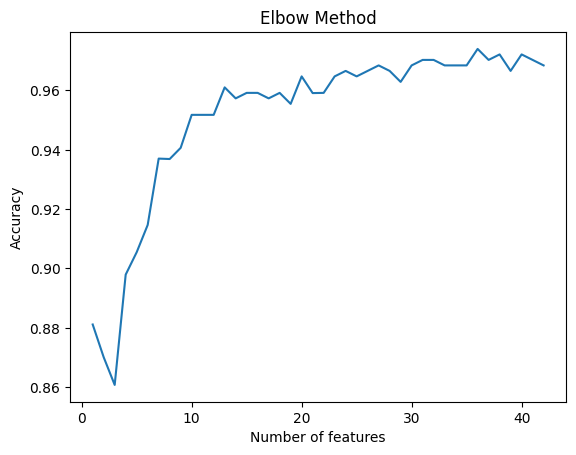


Elbow plot showing classification accuracy as a function of the number of top-ranked features for Set-a

**Fig. SI7**


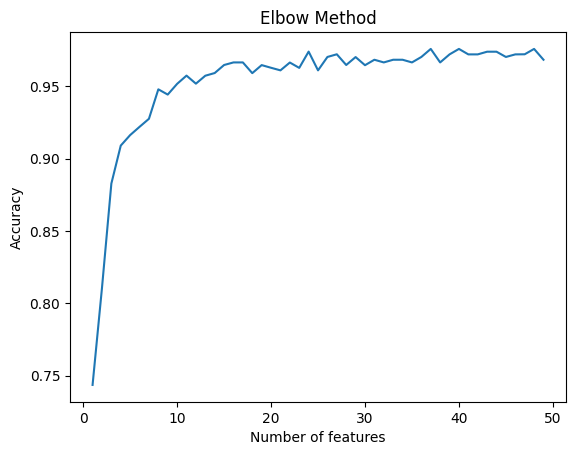


Elbow plot showing classification accuracy as a function of the number of top-ranked features for Set-b

**Fig. SI8**


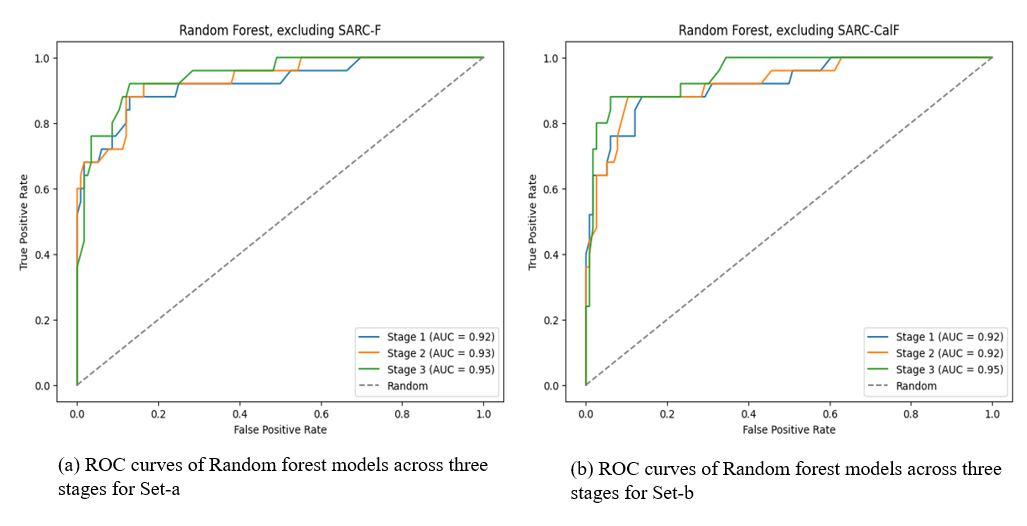


ROC curves of Random forest models across three stages for Set-a (Random Forest, excluding SARC-F), and Set-b (Random Forest, excluding SARC-CalF)
